# Special issue—before translational medicine: laboratory clinic relations lost in translation? Cortisone and the treatment of rheumatoid arthritis in Britain, 1950–1960

**DOI:** 10.1007/s40656-019-0269-7

**Published:** 2019-11-07

**Authors:** Michael Worboys, Elizabeth Toon

**Affiliations:** Centre for the History of Science, Technology and Medicine (CHSTM), Simon Building, University of Manchester, Manchester, M13 9PL UK

**Keywords:** Cortisone, Translational Medicine, Rheumatoid arthritis, Drugs industry, Drug innovation, Non-steroidal anti-inflammatory drugs (NSAIDs), Chronic disease

## Abstract

Cortisone, initially known as ‘compound E’ was the medical sensation of the late 1940s and early 1950s. As early as April 1949, only a week after Philip Hench and colleagues first described the potential of ‘compound E’ at a Mayo Clinic seminar, the *New York Times* reported the drug’s promise as a ‘modern miracle’ in the treatment of rheumatoid arthritis (RA). Given its high profile, it is unsurprising that historians of medicine have been attracted to study the innovation of cortisone. It arrived at the end of a decade of ‘therapeutic revolutions’, kicked off by penicillin transforming the treatment of bacterial infections and ending with hopes of a revolution in the treatment of non-infectious, chronic inflammatory diseases. Despite these studies of cortisone’s introduction, few historians have taken the story forward and considered how cortisone was adopted and adapted into clinical practice. This article tells the longer of how the drug and its derivatives were taken from research laboratories and integrated into clinical practice; what has in recent decades become known as translational medicine (TM). In exploring cortisone’s first decade in Britain, we focus specifically on its role in the treatment of RA. Our approach is always to consider cortisone’s use in the context of other treatments available to clinicians, and at local and national institutional settings. We do not discuss the many other therapeutic uses of cortisone, which ranged for topical applications for skin diseases to the management of cancers, especially childhood leukaemia, nor do we discuss its close analogue ACTH—AdenoCorticoTropic Hormone. We think there are lessons in our study for TM policies today.

Cortisone was the medical sensation of the late 1940s and early 1950s. As early as April 1949, only a week after Philip Hench and colleagues first described the potential of ‘compound E’ at an internal Mayo Clinic seminar, the *New York Times* reported the drug’s promise as a ‘modern miracle’ in the treatment of rheumatoid arthritis (RA) (Marks 1992).^1^ When cortisone reached Britain the following spring, influential *Daily Express* columnist Chapman Pincher (1950) wrote that the drug had prompted ‘a revolution in medical thought;’ he even repeated one doctor’s assessment of a recent medical conference being ‘like a religious meeting, with men popping up all over the house to tell of some seeming miracle.’ Pincher cautioned that cortisone was not a cure and gave only relief from symptoms, but at the same time noted that the drug’s discovery could lead to new treatments for up to twenty other diseases. The Nobel Prize Committee were also impressed, announcing in October 1950 that the prize for physiology and medicine would go to Hench, Edward Kendall and Tadeus Reichstein ‘for their discoveries relating to the hormones of the adrenal cortex, their structure and biological effects’ (Nobel Prize 1950). In Britain, the *Lancet*’s report on this award welcomed the cautious attitude of Mayo clinicians, but even so heralded the drug as ‘the dawn of another new day in therapeutics’ (Anon 1950a, b).

Given these claims, it is unsurprising that historians of medicine have been attracted to development and introduction of cortisone (Kendall 1970; Marks 1992; Hetenyi and Karsh 1997; Slater 2000; Rasmussen 2002; Rooke 2012). However, few historians have taken the story forward to consider the impact and uses of cortisone after the initial excitement (Cantor 1993; Haller 2012). This article tells that longer story of TM, looking beyond cortisone’s first move from the research laboratory to first uses, to focus instead on: (i) how the drug and its derivatives were assessed in trials: and (ii) at the recommendations made to clinicians about its employment in the treatment and management of rheumatic diseases. In terms of the concepts of studies of technological change, this is moving the story on from invention (product creation) to innovation (first use) and then diffusion, though the latter should be understood as involving agency and active processes of adoption and adaption, not passive spread (Freeman 1974; Pickstone 1992, pp. 1–16). An important feature of our approach is to consider cortisone in the context of other therapies, taking cognisance of how these were developed in part due to the stimulus of cortisone’s innovation, and the professional and institutional place of rheumatic diseases in medicine. Our inspiration is the work of Thomas P. Hughes whose work introduced the idea a more holistic, sociotechnical systems approach to histories of innovations (Hughes et al. 1987; Pickstone 1992).

Since 2000, policy and research studies in medicine have reframed these developments in new ways under the banner of Translational Medicine, with four stages: T1—basic research on humans, T2—development of clinical application and trials, T3 clinical implementation and uptake, T4 assessment of outcomes and effectiveness (Sung et al. 2003; Woolf 2008; Fort et al. 2017). In this paper we are principally concerned with T2 and to lesser extent T3. Steven Woolf has emphasised that accomplishing T2 translation from bench to bedside requires a wide variety of disciplinary expertise, including:

[M]astery of the “implementation science” of fielding and evaluating interventions in real-world settings and of the disciplines that inform the design of those interventions, such as clinical epidemiology and evidence synthesis, communication theory, behavioral science, public policy, financing, organizational theory, system redesign, informatics, and mixed methods/qualitative research. (Woolf 2008, pp. 211–212)

We do not think it is useful to reconstruct cortisone’s translation from laboratory chemical to pharmaceutical product in terms of the new concepts of TM. Rather our approach is a social historical one that looks how clinical researchers, consultants, general practitioners, and patients through the 1950s engaged in determining why, when, and how cortisone should, and should not, be used. This approach counters the presentism and teleology seen in many discussions of drug development and uptake, which set out a linear, often seeming inevitable, story of progress, with diffusion and adoption necessarily following innovation. Our study of cortisone’s first decade, offers health policymakers and the medical profession a richer understanding of the complexities and ‘messiness’ of translation and the unpredictability of outcomes.

Examining cortisone’s profile in its first decade in Britain, we focus specifically on its role in the treatment of RA. This was the disease initially seen as most likely to benefit from the new drug—and it is also a disease that has yet to receive much attention from historians of medicine. Britain is also an interesting case because the arrival of cortisone coincided with the early years of the National Health Service and the changes it wrought across the whole of medicine. (Watkin 1978; Webster 1988) This focus means that we do not discuss the many other therapeutic uses of cortisone, nor its close relation adrenocorticotropic hormone (ACTH) which was announced at the same time. Both were used in a wide range of diseases, from topical applications for skin diseases to the management of cancers, especially childhood leukaemia (Gordon et al. 1953; Pearson et al. 1955). Our concern is with the medical reception and evaluation of cortisone trials and the advice given to general practitioners and physicians, especially that from the growing group of specialist rheumatologists. Hence, we mainly use published sources—research articles, trial reports, textbooks, reviews, letters to journals, and popular media.^2^ Corticosteroids were high profile and controversial, hence, these sources contain material that is rich in discursive and divergent views. We have also made some use of the archives of the UK Medical Research Council and US Federal Drugs Administration at particular points.

Our account begins by discussing how RA was understood and treated in Britain in the 1940s, showing how clinicians drew upon a range of physical, surgical, pharmaceutical and even psychological therapies to design appropriate regimes, for a condition that varied greatly between individual patients. The cortisone first used in Britain in early 1950s was imported from the United States. This reliance on external supply meant shortages and high prices, with its use in clinical practice very restricted. Distribution was managed centrally through the NHS and Medical Research Council (MRC) (Cantor 1993; Quirke 2005). By the mid-1950s, when the drug became more widely available, clinical trials, that had also been run by the MRC, showed that its benefits were limited and side effect common. It was no longer the hoped for panacea, rather a niche therapy that was typically one element in combinations of physical and medical measures. By this time, pharmaceutical companies on both sides of the Atlantic had turned their attention to cortisone derivatives such as prednisone, which were said to combine greater efficacy and fewer side effects, and then to different classes of anti-inflammatory drugs—Non-Steroidal Anti-Inflammatory Drugs or NSAIDs. (Hart 1955) Our title queries whether cortisone was ‘lost in translation’? In one sense it was, given its limited use by the late 1950s; however, it had not disappeared. Anything but. We argue that its development and ability to improve the symptoms of patients with RA, despite the risks, stimulated further innovations with corticosteroids and then with NSAIDs. In turn, these developments encouraged innovations in established physical and medical therapies, and all this took place in rapidly changing institutional settings and wider social and health reforms.

## Rheumatoid arthritis before cortisone

The principal pathology of RA is damaged joints, where cartilage becomes inflamed, eroded and degenerates, causing swelling, pain and immobility. The adjective ‘rheumatoid’ indicates that it is a systemic disease, as the humour ‘rheum’ was historically associated with inflammation that could spread throughout the body. The systemic character of RA differentiated it from osteoarthritis, which is localised and largely due to wear and tear to specific joints, and from the less common condition ankylosing spondylitis, which affects the spine. By contrast, RA typically extends from the hands and feet, on to other joints, and then possibly even to affect the heart, lungs and eyes, producing general debility in sufferers. In the most severe cases, the damage produced leads to adhesion and the fusion of joints, all of which causes swelling in surrounding tissues, chronic pain and physical disabilities. These crippling consequences gave RA a fearsome reputation. It was often a ‘disaster for the individual’, and in the public mind the sufferer was imagined as ‘a severely crippled patient in a bath-chair being wheeled along and quite incapable of walking and needing help in all the usual daily activities.’ (Copeman and Mason 1954) There were, however, some ‘hopeful features.’ RA varied from patient to patient in its course and intensity: some sufferers experienced spontaneous remissions without treatment, while many only had some joints affected, and managed to maintain movement thanks to medical and physical therapies and assistive technologies. This left around ten percent of RA patients in Britain experiencing the disease as a severe, incapacitating condition.

State-of-the-art treatments for RA in Britain before the advent of cortisone were described in a lengthy chapter in William Copeman’s *Textbook of Rheumatic Diseases*, published in the autumn of 1948. The chapter was written by Stanley Davidson, from Edinburgh Royal Infirmary. (Davidson 1948, pp. 120–143; Robson 2004) In the 1930s, he had been a founder member of the Empire Rheumatism Council (ERC), a charitable body that funded research and had become the dominant political force in British rheumatology. Led by the influential Lord Horder, its remit had expanded from research to all aspects of the disease and to health policy more widely (Cantor 1992, 1993). It was to play an important role with cortisone in Britain.

Davidson began by stating that options were constrained because the cause of the disease was unknown. However, he was clear that doctors could help sufferers by mitigating symptoms with both general and specific measures, in a socio-technical system of institutions (spas, out-patient clinics, physician practices), with varied doctor-patient relationships, and multiple types of intervention (baths, physiotherapy exercises, manipulations and machines, drugs, occupational therapy).

Davidson’s views on aetiology were typical in suspecting that the onset was triggered by infection, a claim supported by the experience that sufferers were known to benefit from regimes similar to those used for ‘other chronic diseases of infective origin.’ His assumption was that infection had produced persistent ‘disordered immunological processes,’ which in turn led to inflammation and stiffness, along with fatigue that was exacerbated by persistent pain. Davidson was typical in making an explicit parallel with tuberculosis, one form of which affected the bones and joints and some treatments echoed sanatorium regimes of rest, fresh air, sunshine, cheerful surroundings and a full diet.

Davidson set out the accepted figures for prognosis in a typical patient population: ‘if treated early and efficiently’ a quarter of those treated could be restored to normal health, while ten percent would become ‘completely crippled’ despite adequate treatment. The remaining sixty-five percent of patients could be expected to show ‘functional improvement’ varying ‘from mild to good.’ In sum, the situation was, he suggested, better than generally believed, but interventions needed to be early and given with a ‘a hopeful outlook.’ Pain relief was the cornerstone of treatment, and maximum relief required a careful balancing of analgesics such as aspirin, phenacetin, codeine and barbiturates, and ensuring patient compliance. Next in importance was physiotherapy, which included both the local manipulation of joints by trained professionals and a programme of general exercise that would allow the patient to maintain overall mobility. Complementing pain relief and physiotherapy was occupational therapy, which would occupy the mind of the sufferer and counter possible adverse psychological factors. While Davidson admitted that there were no ‘controlled investigations’ to support the idea that emotional strain could lead to arthritis, he nonetheless suggested that ‘the clinical impressions of experienced physicians must not be discarded lightly.’

When it came to specific treatments, especially drugs that were claimed to have specific therapeutic effects, Davidson was scathing: ‘Of the hundreds of remedies which have been recommended in the treatment of rheumatoid arthritis, the majority are useless’ (Davidson 1948, p. 137). He was far from alone in his scorn. In a similar review of treatments published that same year, the head of London Hospital’s Department of Physical Medicine, William Tegner, likewise observed that ‘There is as yet no treatment for RA that can compare with the specific action of vitamin D in rickets or of sulphonamides and penicillin in acute bacterial infections’ (Tegner 1948, p. 470). It was not, however, for lack of trying. Davidson offered a list of specifics that had previously enjoyed clinical support—‘arsenic, iodine, sulphur, guaiacol, ortho-iodoxybenzoic acid, histamine, bee venom and the thiosianime group of drugs’, while Tegner offered a similarly varied but entirely different list: ‘bismuth, copper, protein shock, jaundice, splenectomy, erepsin, ‘Ertron,’ (vitamin D) spinal pumping, and the blood of pregnant women.’

The only specific drug treatment that Davidson and his peers endorsed for treating RA (as opposed to simply relieving the pain associated with it) was chrysotherapy, the injection of gold salts (sodium aurothiomalate, branded as Myocrisin). The modern use of gold compounds in therapy began with Robert Koch, who tried them as antibacterial agents with tuberculosis in the late 1880s. As Rodnan and Benedek (1970) have shown, their use persisted until the advent of antibiotics in the 1940s, and the presumed similarities between tuberculosis and RA prompted trials in the early 1930s by French physician Forestier (1932). Gold treatment for RA was taken up across the world, although with more enthusiasm in Europe than in the US. One of the largest studies was undertaken by Stanley Hartfall and colleagues at the Leeds Public Dispensary and Hospital in the mid-1930s (Hartfall et al. 1937a, b). Patients were given repeated courses of treatment consisting of a dozen weekly injections, each followed by at least a 3-month interval away from treatment. Of 750 RA patients, eighty percent showed ‘striking improvement,’ with a relapse rate of twenty percent. Toxic reactions occurred in forty percent of cases, but Hartfall and his colleagues found these ‘as a rule did not contraindicate further treatment.’ In 1939 Davidson, who was then on the ERC’s Chemical Sub-Committee of its Scientific Advisory Committee proposed ‘an investigation of gold therapy in RA, with a carefully planned system of controls and provision for independent assessment of clinical results and of statistical check’, to be undertaken across many centres. (Horder 1940) This was not a randomised controlled trial as developed later in the 1940s, though it did have similar features. The proposed trial was first delayed and then truncated by the Second World War, though the results from a single centre (Glasgow), with 103 patients, was eventually published in June 1945. Thomas Fraser (1945), who led the study, reported 82% of patients on myocrisin improved as against 45% controls However, 75% of patients given the drug had toxic reactions, though most were said to be mild; there were no deaths. Fraser concluded:

Gold salts are of value in the treatment of rheumatoid arthritis, particularly when used in conjunction with general, physiotherapeutic, and orthopaedic measures. On account of their toxicity they should only be employed by those with experience in their use. (Fraser 1945, p. 75)

Lord Horder, then chair of the ERC, made no mention this study in his Annual Report for 1945 and the tenor of his remarks was there was nothing new to say on treatment (Horder 1945).

Davidson’s views in 1948 were similar, namely that ‘gold salts must be considered merely a part of—and not a substitute for—a general plan of treatment carefully arranged for each particular patient’ (Davidson 1948, p. 138). However, he was more cautious than Fraser, stating first and foremost that patients given gold salts needed to be closely monitored for adverse effects. Moreover, that smaller doses over longer periods gave the optimum balance of benefits and risks. His figures, based on published reports, were that proportion of patients developing toxic effects was lower than Fraser’s at 20–50%, though he reported that there were severe effects in 5% of patients, with 1% fatalities. Davidson’s text carried the sense that gold therapy was dangerous, especially in the wrong hands. Adverse reactions, including ‘gold hepatitis’, blood in urine, and skin eruptions were unpredictable, so careful counselling of patients was essential, and treatment had to be halted at any toxic signs (Freyberg 1950). None the less, he concluded that with clinical acumen, gold therapy could benefit the general health and local manifestations of arthritis in 80% of patients, and that it was unsuitable or dangerous in just 10%. In his review Tegner was ambivalent, writing that ‘Chrysotherapy is risky but is worth trying after all other methods have failed’ (Tegner 1948, p. 472).

## Cortisone

Philip Hench was already a celebrity rheumatologist when he announced ‘compound E’ in April 1949. He had been appointed head of the Mayo Clinic’s rheumatology service in 1928 and was a founding member of the American Association for the Study and Control of Rheumatology (Stecher et al. 2010). From 1937 to 1948 he wrote the most influential annual literature review of the field, published in *Annals of Internal Medicine* (Stecher 1985). Hench was held in high esteem by rheumatologists in Britain, having been awarded the Heberden Medal in 1942 for his investigations on remission of RA in patients with jaundice (Moll 1987, p. 175). In April 1947 he had published an influential review of chrysotherapy, with similar judgments and advice to those of Davidson (Hench 1947). Hench was in Britain in October 1948, at the invitation of the Heberden Society, delivering public lectures on ‘Psychogenic Rheumatism’ and ‘Current remedies for rheumatoid arthritis’ (Hench 1948). The *British Medical Journal* (*BMJ*) reported that Hench opened by saying ‘he had nothing striking or wonderful to bring before his audience’ and spent most of the lecture criticising current remedies. He began by stating that in his experience, general supportive measures led to 15% of patients being cured and a further 35% improved. He then argued that this could be raised to 75% with special measures such as gold therapy. However, he warned physicians of the range of remedies of dubious value, showing a slide in which a remedy was listed for every letter of the alphabet, even including Z—“zero-therapy”(do nothing). The lecture ended with ‘mention’ of remissions induced by spontaneous jaundice and pregnancy, where over 90% of patients improved. In a clear trailer for soon to be announced ‘compound E.’ he argued that ‘there was an unrealized potential in these conditions’ to make RA ‘potentially reversible’ and eventually ‘therapeutically controllable.’

Hench’s Oration to the Heberden Society (1949), given on 15 October 1948, was eventually published in the *Annals of Rheumatic Diseases* in June 1949, 2 months after the announcement of ‘compound E’ as a treatment for RA. It was accompanied by an Editorial on ‘compound E,’ on which Hench and colleagues had published in the April edition of the *Proceedings of the Staff Meetings of the Mayo Clinic* (Editorial 1949a; Hench et al. 1949). The first British medical assessment of ‘compound E’ came the following month, after the return of the ERC-led delegation to the International Congress of Rheumatology in New York (ICR 1949). The tone of the report in the *BMJ* was cautious (Anon 1949b). It quoted Hench’s remarks that ‘compound E’ was ‘the end of the beginning’ and that more research was needed on its production and administration. Limited supplies, due to the difficulties and expense of its extraction from the ox bile, made ‘inappropriate now the use of the term “treatment” except in an investigative sense.’ The following week the *BMJ* published an editorial that noted that, because of ‘compound E’, there was a new optimism in rheumatology and, to reinforce the point, published four articles on progress in different forms of rheumatism (Editorial 1949b). In the weeks after the Congress, a group of British delegates visited the Mayo Clinic and were shown patients with RA who had become ‘completely symptom-free’ after being given the drug. They also learned of occasional undesirable side-effects, which reinforced the view that what was needed was ‘well-controlled experimental work for some time to come’ (ICR 1949, p. 155).

In October 1949 the British Rheumatic Association discussed ‘compound E’ (BRA 1949). William Copeman, who was in the group that had visited the Mayo Clinic, suggested that ‘compound E’ would eventually rank with insulin and penicillin as great medical breakthroughs, though it was still not a ‘practical treatment’ because of the problems of supply. One daily dose of the drug required bile from 40 oxen carcasses. Oswald Savage also spoke and implicitly contrasted ‘the excitement which this discovery had aroused in America,’ with the reaction in Britain where there were no patients with miracle improvements in symptoms to attract the popular press.

Doctors in Britain followed events across the Atlantic very closely (Editorial 1949c). In January 1950, John McNee, Regius Professor of Medicine at the University of Glasgow, reported on his recent 3-month stay and was obviously affected by the new mood, claiming that ‘something quite new and fundamental in medicine and therapeutics had been discovered, with far wider possibilities and implications far beyond the boundaries of the chronic rheumatic diseases’ (McNee 1950, p. 114). The other diseases he listed were: ‘acute rheumatic fever, chorea, gout, chronic ulcerative colitis, lupus erythematosus diffusa, scleroderma (with oesophageal involvement), asthma, lymphadenopathies (including lymphosarcoma and Hodgkin’s disease).’ On ‘compound E’ and ACTH, McNee reiterated the now familiar accounts of ‘rapid and intensely dramatic effects’ in patients with RA, observing that though the drug’s effects were like turning a tap on and off, many courses of treatment led to ‘lasting improvement over a number of months.’ However, there were side effects to consider. According to McNee, ‘none was severe’ and most ceased with the end of the treatment. The commonest was Cushing’s syndrome, which he termed relatively benign, while the most severe were ‘persistent hypertension, glycosuria and even true diabetes, acute spread of tuberculosis, and violent mental disturbance.’

McNee also dwelt upon supply and cost issues, and the search that was now on for new sources of cortisone and for other compounds that would give the same or better results (Cantor 1993). In July 1950, George Pickering, a senior physician at St Mary’s London, was already looking forward to ‘compound E’ and A.C.T.H. being displaced with ‘by something whose effects are more narrowly confined to the fundamental disturbance in these diseases’ (Pickering 1950). These hopes reflected the optimism about new pharmaceuticals that was current across medicine and society at this time, fuelled primarily by the series of innovations with antibiotics (Bud 2009).

The MRC had taken an immediate interest in ‘compound E’ anticipating a similar pattern of centrally-led development and distribution as seen with insulin in the 1920s (Liebenau 1989). A committee was set up in 1949, which in 1950 merged with the Nuffield Foundation’s Clinical Trials Committee. Their first task was to oversee work on the gift by Merck of 1 kg of ‘compound E’, which was now increasingly referred to its brand name cortisone (Medical Correspondent 1950). The first trial was led by William Copeman at the West London Hospital and began with just five RA patients (Copeman et al. 1950). Patients were drawn from two hospitals and other steroids were investigated: androstenedione, dehydro-iso-androsterone, progesterone, pregnenolone, and pregadienolone. The results were published in October 1950. The alternative steroids showed no benefits, however, ‘the dramatic clinical effects of cortisone were immediately obvious.’ Given further supplies, Copeman’s team were able to extend their trial with 20 patients over 3 months, with the same protocol. They published the results in February 1952 (Copeman et al. 1952). The challenge was to find ‘a suitable method of administration which will suppress the disease indefinitely and enable the patients to resume useful and independent lives, free from unpleasant side-effects.’ Every patient improved, though care had been taken because of variation in individual patients between ‘the suppressive dose and the “ toxic” dose.’ In one patient’s case, the side effects were so severe the treatment had to be ended. However, the verdict overall was that the benefits outweighed the risks, and that perhaps too much significance was being attached to ‘mild side effects;’ which the authors compared to ‘a house-proud woman over conscious of a speck of dust which the visitor has not noticed.’

By this time Copeman had become *the* British authority on the use of cortisone in rheumatic diseases. He wrote one of the *BMJ*’s ‘Refresher Courses for General Practitioners’ on RA, published in June 1952 (Copeman 1952). He covered all aspects of the disease: aetiology, pathology, symptom management and active treatment. He left cortisone and ACTH to last, stressing that even Hench and his co-workers still spoke about the drugs as experimental treatments and that it would likely be many years before they were available to general practitioners. After discussing benefits and risks, Copeman ended by suggesting that the drugs ‘will take their place as a very valuable medical treatment in selected cases’, but that ‘It is unlikely, however, that their use will ever entirely eliminate the need for the use of the other less specific types of treatment.’

Copeman’s public view had changed little by December 1953 when he published an update on the 21 patients still in the MRC-Nuffield trial. He remained positive, although it was clear that he saw cortisone and ACTH as additional weapons in the rheumatologist’s armoury, to be used cautiously and selectively (Copeman 1953). At this time, the greatest difficulty facing doctors was patient selection due to the limited supply and cost of the new drug. In early cases, Copeman and colleagues continued to recommend rest and that ‘other measures such as gold’ be given a reasonable trial, because once cortisone was started, it ‘will probably have to be continued for a very long and perhaps indefinite period and the disease will be suppressed and not cured by it.’

Two developments ensured that cortisone was not the only innovation in the treatment of RA. The high profile and presumed potential of cortisone led pharmaceutical companies to search for compounds with similar properties, easier manufacture, and lower cost. Second, there were calls to apply the rigours of the new clinical trials, developed with antibiotics, to all therapies new and old. In fact, Austin Bradford Hill, who had pioneered the development of randomised clinical trials (RCTs) was in cortisone trials from an early stage (Copeman et al. 1950). David Cantor (1992) showed that British rheumatologists were unanimous that cortisone’s value had to be seen against the other therapies that they used and with which there continued to be innovations. The most promising new drug was Butazolidin (phenylbutazone), launched by Geigy as a specific for rheumatic diseases in 1951 (Worboys and Toon 2018). Phenylbutazone (or ‘bute’ as it was sometimes called) was an effective analgesic, but rheumatologists disagreed about its anti-inflammatory action and there were reports of severe side-effects (Hart and Johnson 1952). In 1953, phenylbutazone came under intense scrutiny because of the number of reports of adverse reactions in patients (Leading Article 1953). Specialists’ verdict was that the drug could be useful, mostly in ankylosing spondylitis, but that its potential for causing life-threatening risks meant it required very careful monitoring (ERC 1953; RSM 1953; Currie et al. 1953).

Innovations with gold therapy continued and the treatment enjoyed strong support from clinicians. However, there was a growing demand for it to be subject to the new RCTs. The challenge was taken up Ralph Peebles Brown and R Currie and in 1953 they published the results of a trial undertaken with 220 out-patients at the Rheumatism Unit at the Glasgow Royal Infirmary (Peebles Brown and Currie 1953). They had no access to cortisone, so gold therapy was compared with copper, arsenic, aspirin and physiotherapy, with saline as the no treatment control. They concluded that ‘a single course of treatment with gold salts [was] no better than those obtained by the other methods described.’ Their findings were strongly criticised by Jacques Forestier, the long-standing champion of the gold therapy. He responded that gold salts, though not a panacea, had stood the test of 20 years of clinical experience, but that ‘they would not remain in use if they did nothing more than saline injections’ (Forestier 1953).

By 1954, with more trial data and practical experience to hand, British rheumatologists felt able to speak authoritatively on cortisone, and even to challenge the dominant wisdom according to American researchers. This challenge grew partly out of British rheumatologists’ realisation that even within American medicine, views of cortisone were mixed, as well as the recognition that in some ways the culture of American medical research and practice differed considerably from their own.

One especially keen observer was John Glyn, who offered his British colleagues a lengthy report on his experience as a Fulbright and Masonic Fellow in New York in 1952 and 1953 (Glyn 1953; Liyanage and Seifert 2006). On cortisone and ACTH, he found ‘The discrepancies of opinion about these drugs in different centres startling.’ Indeed, he revealed, there was actually an ‘amazing cleavage of opinion’ in the American Rheumatism Association’s study of the new drugs, which he attributed to biases in different centres and there being no agreed criteria for success. In contrast, the British testing was centralised due to the NHS, the dominance of the MRC, and limited supplies of the drugs. Also, Glyn suggested that clinical evaluations in Britain, for what he called ‘political reasons’, stressed functional improvement and by implication the ability to work and not claim benefits. In the United States, he suggested, the emphasis was pathological not social, which meant ‘the undoubted evidence of disease progression during cortisone therapy classes it as a palliative drug and that it is far too dangerous a weapon to be used in this way.’ At the Mayo Clinic, he learnt that Hench felt that the drugs ‘have been grossly misused,’ with high dosages used to produce dramatic, but inherently dangerous results. Best practice was moderate dosages, adjusted to balance symptom relief and the avoidance of significant side effects. Glyn concluded that a similar disagreement shaped American thinking on phenylbutazone, with ‘confusion reign[ing]’ as to the drug’s value and toxicity.

A key reason for the confidence of British rheumatologists was the publication on 29 May 1954 of the Joint Committee of the MRC and Nuffield Foundation comparing cortisone and aspirin in the treatment of early cases of RA that found little difference in outcomes (Joint Committee 1954). The drug had been given to selected elite clinicians in rheumatology clinics, who followed centrally produced protocols, based on the new randomised clinical trial (RCT) standards. An important element in the protocol was that all patients continued to have physiotherapy, use splints and benefit from other physical methods, showing that, even in cutting edge trials, innovations ‘old’ and ‘new.’

The wording of the report on the trial was that ‘For practical purposes … there appears to have been surprisingly little to choose between cortisone and aspirin in the management of the 61 patients in the early stages of rheumatoid arthritis.’ The results had been first presented to the Joint Committee of the MRC and Nuffield Foundation on 26 January 1954, not by a rheumatologist, but by Bradford Hill (NA FD 1/8059, J.C. 12 (Minutes); Hill 1954). He reported that ‘In both groups changes in joint tenderness, range of movement, mobility and functional capacity followed a similar pattern.’ The main difference was that patients on cortisone showed more complications in the last 6 months of the one-year trial. At the meeting, one member, the renowned biochemist Professor Charles Dodds, suggested the government should now stop investing in new ways to produce cortisone (Cantor 1993), but the Committee rejected this. The minutes for the meeting indicate a strong divergence of opinion:

Copeman was very much up in arms at any suggestion that continuous treatment of cortisone might be abandoned. He criticised the lines of the trials on minor points and suggested that results of early cases might not be applicable to other types: publication of a report might be misleading. (NA FD 1/8059, J.C. 12 (Minutes)

This intervention was apparently sufficient to convince Committee members that the publication of the results of the trial would need to be carefully handled. (NA FD 1/8059, J. C. 12, paras 188-189).

But another member of the Committee, Ian Duthie, broke ranks and revealed the findings of a preliminary report at a meeting of the Royal Society of Medicine that very evening. He only described the results at 3 months, not the full year, but nonetheless concluded that ‘long-continued administration of cortisone to patients suffering from RA was not to be recommended until complications which cannot be foreseen or prevented at present can be avoided with certainty’(Duthie 1954). In support, he mentioned a recently published study by Sheffield rheumatologists Harry West and George Newnes, who had also concluded ‘that long-continued cortisone therapy for rheumatoid disease is not to be recommended’ (West and Newnes 1953). The following speaker at the meeting was Copeman. He disagreed with Duthie, arguing that the trial had many faults and the positive outcomes enjoyed by the 20 patients in the long running trial at the West London Hospital were a more reliable guide for future practice (Copeman 1954).

The Joint Committee Report was eventually published in the *BMJ* on 29 May 1954, offering its now famous conclusion on the seeming equivalence of cortisone and aspirin. In the same journal, just a fortnight previously, Copeman too indicated his dissent by rushing out the more positive results of his study on prolonged administration of cortisone at the West London Hospital (Savage and Copeman 1953; Copeman et al. 1954). He felt they were particularly significant because they involved extended clinical experience and demonstrated that under careful specialist supervision, ‘previously incapacitated … patients can be restored to and maintained in relatively normal activity.’ The edition of the weekly *BMJ* between Copeman’s article and full Joint Committee Report, carried a letter from Dudley Hart and colleagues reporting their unit’s results in the cortisone-aspirin trial (Hart et al. 1954). In another pre-emptive defence, they emphasised the positive. Hart reported that cortisone gave ‘increased relief from those annoying and distressing symptoms which, while hardly appearing in statistical surveys, do make the lot of the rheumatoid sufferer somewhat easier.’ The *BMJ* Editorial that accompanied the Joint Committee Report focused on the difference between its results and reported by Copeman, Duthie and others, concluding that with little to choose between benefits, the less severe side-effects of aspirin made it the treatment of choice for RA. (Editorial 1954)

The results of the cortisone-aspirin trial soon found their way into the popular press. One headline in the *Daily Express* was that ‘Aspirin May Be Best,’ while the *Manchester Guardian* led with ‘Aspirin Equals Cortisone’ (Anon 1954a, b) In the correspondence that followed in the medical journals, John Glyn (1954) worried that the ‘glaring publicity’ given to what he regarded as the flawed conclusions of the Report, ‘will undoubtedly have considerable medical and political repercussions.’ His complaint was that statistics had overridden clinical experience and that ‘the pendulum away from therapeutic empiricism has swung too far.’ Glyn claimed that

Perfect statistical techniques are not possible when dealing with biological material, nor is it possible at the early stage at which the Medical Research Council generally formulate their trials for them to lay down the optimum regimes which are not liable to subsequent criticism.

Bradford Hill (1954) answered Glyn the following week, focusing on general principles rather than the cortisone trial. He disputed any characterization of trials operating with statisticians ‘as dictators and the “experienced, reliable and unbiased clinicians” as their puppets.’ Rather, he maintained, the statisticians and the clinicians had collaborated at all stages.

Despite the efforts of Copeman and other to defend the benefits of cortisone, the press reaction shaped public and to an extent non-specialist medical views (Anon 1955, 1956). If cortisone was no better than aspirin and had risks, and was expensive to boot, why use it? The Second Report of the Joint Committee of the MRC and Nuffield Foundation in September 1955, based on a further year of their trial, again concluded ‘that for practical purposes there has been remarkably little to choose between cortisone and aspirin.’ (Joint Committee 1955) Indeed, when improved supplies finally made it possible for general practitioners to prescribe the drug in December 1955, an editorial in the *BMJ* was lukewarm. (Editorial 1955c) There was little mention of the drug’s benefits, and a caution that symptoms were only suppressed for as long as the drug was being taken. Before embarking on use of cortisone, doctors were told to ask three questions. First, was there a ‘less dangerous drug’ that would be just as effective? Aspirin and, no doubt to the surprise of many, phenylbutazone were the alternatives offered. Second, did the patient have an existing condition that would be exacerbated? The most immediate and serious were latent tuberculosis, heart failure, or gastric ulcer. Third, the doctor had to consider when and how to end the treatment, for with patients of needing the drug long-term or at a high dosage, there was ‘a formidable list’ of complications that included psychoses. The editorial ended thus: ‘Powerful drugs such as cortisone increase the physician’s responsibility and his power, not only to do good, but, by inadvertence, harm’ (Editorial 1955c).

## And after

On 12 November 1955 the *Lancet* had also carried an editorial on the wider release of the drug entitled ‘Cortisone and After’ (Editorial 1955a). The author contended that the days of cortisone were numbered now that two new corticosteroids, prednisone and prednisolone, promised to be more effective and less toxic, had been introduced. An earlier report on the drugs was undertaken by Frederick Dudley Hart and his Westminster Hospital colleagues, which appeared in the same issue of the journal had prompted the editorial (Hart 1955). Hart’s conclusion was that ‘In a short-term study prednisone and prednisolone proved in 1/4–1/8 dose more effective than cortisone in relieving the symptoms and reducing the signs of rheumatoid arthritis.’ None the less, the accompanying editorial was cautious, ending by stating that ‘Experience with cortisone in the treatment of RA will make clinicians wary in the use of prednisone and prednisolone’ (Editorial 1955b).

The following week the journal carried another editorial on cortisone and an article by Richard Bayliss of the Westminster Hospital on ‘The Use and Limitations of Cortisone in General Practice’ (Bayliss 1955). This editorial accepted that cortisone had ‘established itself as a valuable drug’ and deserved to be made more widely available, but cautioned that ‘a heavy responsibility rests on the general practitioner who uses it, for it is expensive and it has potentially dangerous side-effects’ (Editorial 1955b). Bayliss had produced a hospital doctor’s guide for the uninitiated and his advice came with the warning that: ‘What is written today may well need modification tomorrow, and if this account of the uses of cortisone errs on the side of conservatism it is because I have deliberately chosen to avoid speculative claims.’ In fact, he began his guide by setting out ‘Side effects’ and ‘Contraindications’, and only then moved on to deal with its therapeutic use, not just with rheumatic conditions, but also skin, respiratory, eye, nephrotic and blood diseases. With RA, he suggested that cortisone should be turned to when aspirin was ineffective and when patients had active inflammation. Bayliss was also clear that cortisone was being superseded and that, ‘We are on the threshold of a new and important era in the control of some hitherto fatal or disabling diseases.’

Over the next 5 years, chemists, working mainly in American pharmaceutical companies, produced a succession of new compounds that they claimed were more efficacious and less harmful than existing corticosteroid drugs. The most important drugs were triamcinolone, produced by both Lederle (branded Aristocort) and Squibb (Kenacort) in 1957; methyl-prednisone (Medrol) and fluprednisolone (Alphadrol) by Upjohn in 1957; and dexamethasone (Decadron) by Merck in 1958 (Benedek 2011; Hogg 1992). However, Schering’s prednisone (Meticorten) and prednisolone (Meticortelone) while the first, proved the most enduring. All these drugs were developed by adding and subtracting different chemical groups to the cortisone molecule, or its close analogues. In some new drugs, molecules were changed ‘by design’, while in other cases researchers relied on serendipity ins what was termed ‘chemical roulette’ (Dunlop 1978). Pharmaceutical companies were also competing to find more efficient and economical ways to make these drugs, using synthetic methods and what would now be termed biotechnology.

The business of finding, testing, synthesizing, upscaling to production, marketing and selling these new drugs became controversial in the United States in 1957 because of alleged price-fixing and profiteering. This led to investigations by the Senate Antitrust and Monopoly Subcommittee, led by Senator Estes Kefauver, which began his interest in drug regulation, and in 1962 the famous Kefauver-Harris Amendments (Nossiter 1959; Scroop 2007; Tobbell 2011; Greene and Podolsky 2012; Greene et al. 2016)

In Britain the assessment of the new corticosteroids remained with the Joint Committee of the MRC and Nuffield Foundation. As early as 1956, they set up trials to compare prednisone and cortisone. (Joint Committee 1957) Amongst patients already in their trials, 33 patients were continued on cortisone therapy and 35 transferred to prednisone. The results were clear.

Throughout the year of the trial the patients remaining on cortisone showed, on the average, no material change for better or worse. The prednisone group, on the other hand, showed improvement in the following characteristics—strength of grip, blood sedimentation rate, haemoglobin level, general capacity, and disease activity. At the end of the year the disease was judged to be inactive in five of this group but in none of the patients on cortisone. (Joint Committee 1957, p. 201)

Improvements were greatest in the early months and over the trial period a number of side effects were reported, which were attributed to the higher dosage allowed with prednisone. A follow-on trial that compared prednisolone and aspirin over 2 years also had positive results (Joint Committee 1959).

[T]he administration of prednisolone instead of, or in addition to, analgesics, such as aspirin, to certain patients with rheumatoid arthritis for a period of 2 years will, on average, improve their functional capacity and general well-being and reduce the incidence of erosive joint damage. (Joint Committee 1959 p. 185)

Moreover, though side effects were again present, they had proved manageable by reducing the dosage and retaining some benefits. In the following year, the Committee reported on a further year’s trial, the improvements for patients had been maintained, ‘though to slightly lower degree.’ (Joint Committee 1960)

By the end of the 1950s, leading Californian rheumatologist Edward Boland, who had been one of the five participants in the early cortisone trials in the United States, wrote influential reviews of the ‘relative therapeutic efficiencies’ of chemically modified adreno-corticosteroids in RA (Boland 1959; 1960). In both reviews he was clear that new analogues had superseded the once-miraculous cortisone. The argued that the impact of the new steroids as a class, in retrospect, had been evolutionary rather than revolutionary. In his 1959 review, Boland stated that ‘Steroids should be reserved for carefully selected cases: those with active and potentially reversible disease.’ He continued, that they should only be used after conservative therapy had been given a fair trial and then:

Steroids should not be prescribed to the exclusion of other measures. A sound comprehensive program should be employed concomitantly; this would consist of well-regulated rest and exercise, properly balanced diet, avoidance of undue emotional stress, physiotherapeutic procedures and graded muscle exercises, careful handling of affected joints with proper supports and splints, occupational and rehabilitative measures, and simple analgesics. (Boland 1959, p. 888)

Ending therapy needed careful management too: ‘treatment must be gradually discontinued or “tapered off’’—not suddenly withdrawn. ’The physician’s goal, as ever, was to balance benefits and risks: ‘to promote and uphold that degree of disease suppression that is feasible with dosages that are well tolerated on a long-term treatment basis.’ In his review a year later, Boland’s verdict was that results with the new corticosteroids had been mixed.

Some possess advantages in one respect or another, but none is divested of the major deterrent features of corticosteroid therapy, and some produce peculiar objectionable reactions of their own. Because of a lack of published comparative data and misleading promotional pharmaceutical advertising, physicians have been confused regarding the relative merits, special properties, and tendencies to unwanted reactions of these newer preparations. (Boland 1959, p. 888)

With experience and the use of clinical acumen, informed by trials and case reports, rheumatologists could adopt the new drugs selectively, finding them niches in their multi-strand therapies, adjusted to individual patients and their disease.

In Britain in 1960, Dudley Hart (1960) wrote a similar review for British doctors and with the same verdict. He wrote that ‘There is relatively little to choose between the various steroids available for therapy,’ except in relation to side effects. The main advantage of the newer drugs was that lower doses gave similar disease suppression: ‘25 mg. of cortisone equals 20 mg. of cortisol, 5 mg. of prednisone or prednisolone, 4 mg. of triamcinolone or methyl prednisolone, and about 0.85 mg. of dexamethasone.’ However, overall there was little gain as ‘Claims of diminished incidence of side-effects of one sort or another are made for each new steroid as it arrives, but clinical experience usually shows up such claims as wanting in whole or in part.’ Edward Boland, in a lecture in London in September 1961, repeated his earlier message: ‘Some of the analogues exhibit qualitative differences in one or another physiological action, but the major deterrent features of the corticosteroids are shared by all of them’ (Boland 1962).

In the Third Edition of Copeman’s *Textbook of Rheumatic Disease*, published in 1964, Ian Duthie wrote the chapter on rheumatoid arthritis. He was critical of both old and new therapies.

In recent years the results of adequately controlled clinical trials have shown that many remedies popular in the past are without effect on symptoms or the course of the disease. It has also been shown that although a number of drugs can make a useful contribution to treatment, none that proved predictably effective or harmless. Remissions induced by these have not been sustained and the ultimate course of the disease does not appear to have been significantly altered for the better and may even have been made more severe. An example of this, seen only too frequently, is the markedly increased severity of both arthritis and constitutional symptoms which may follow the injudicious withdrawal of corticosteroids. Fatal complications have also been induced by other remedies such as gold and phenylbutazone, which were given in good faith and in the absence of known contraindications. If follows that simple conservative measures should be used in the first place with the great majority of patients. (Duthie 1964, p. 208)

Duthie was direct on cortisone and its analogues. He wrote that ‘It is clear that corticosteroids have a distinctly limited role in the treatment of rheumatoid arthritis’ and that derivatives ‘share the major deterrent features of earlier corticosteroids.’ He only recommended drug treatment for patients with ‘disease of longer duration and in whom activity has not been controlled by an adequate trial of … conservative measures.’ The drugs discussed were, in order: aspirin; phenylbutazone and oxyphenylbutazone; corticosteroids and ACTH; chloroquine and hydrochloroquine; and gold. It might have surprised many readers that gave most attention to gold therapy, concluding ‘In spite of its obvious limitations and dangers, the majority of experienced observers consider that chrysotherapy is of value in the treatment of rheumatoid arthritis’ (Duthie 1964, pp. 221–228).

In 1964, William Copeman, by then head of the ERC, summed up the position thus:

It is clear that corticosteroids have a distinctly limited role in the treatment of rheumatoid arthritis. Their use should never be considered until conservative measures applied over an adequate period of time have failed to control progressive and crippling disease. (Copeman 1964, p. 223)

Thus, the wonder drugs of the 1950s were second line therapies, and then that had to be closely monitored and of limited duration due to safety concerns.

In the Introduction of a book entitled *Steroid Drugs*, published in 1962, the editor, Norman Applezweig, suggested that ‘The Cortisone Era’ was over and that modifications of its molecule had produced drugs with improved properties. (Applezweig 1962) However, a bigger change was imminent. In 1963, a new class of drugs was announced: ‘non-steroidal anti-inflammatory agents’ (NSAIDS) (Shen et al. 1963). The first such drug was indomethacin, produced by Merck, Sharp and Dohme, with the results of the first British case reports published in October 1963 (Hart and Boardman 1963, 1965). The companies marketing these drugs claimed that they had better and wider anti-inflammatory properties, plus they had anti-pyretic effects. Crucially were without the range or severity of side effects as still associated with steroids. This property was present in the oddly negative naming—the drugs were defined by the fact that they were not steroids—indicating how problematic steroids had become.

## Conclusion

The history of cortisone’s first decade can be read as showing that as a treatment for RA was ‘lost in transition’ twice. First, in the early 1950s, though rapidly taken from bench to bedside in trials with a select group of clinicians, its spread into hospital and general practice was very limited. Its adoption and uptake were checked by shortages of supply, high cost, uncertainties over dosage and worries about side effects. Second, from the mid-1950s and paradoxically just as the problems of supply and price were solved, pharmaceutical companies had begun to produce new and different variants of cortisone, in a new class of drugs—the corticosteroids (Bunim et al. 1955). And, by the early 1960s, these alternatives were supplemented by a new class—NSAIDs.

Yet, the very naming of NSAIDs as ‘Non-Steroidal’ shows that cortisone had not been lost. It continued to define, albeit negatively, a pharmacologically-based, anti-inflammatory therapeutic strategy for the treatment of RA. Moreover, it was one that accepted that such drugs offered relief of symptoms rather than a cure. The initial hope of a’miracle cure’ for rheumatic diseases had had the effect of stimulating therapeutic innovations with established treatments as well as new pharmaceuticals. Thus, clinicians had a greater range of combinations of physical and medical treatment, even setting up RCT comparisons. The ‘messiness’ of translations identified by Steven Woolf was evident in the ways that cortisone and its derivatives were typically used in combination or alternatively with physical treatments, physiotherapy, splints, etc. and other chemotherapeutic methods.

Our discussion of cortisone has shown the limitations of focussing on a single innovation and the advantages of the socio-technical systems approach of Thomas Hughes. For while over the 1950s as a whole, cortisone was little used in the treatment of RA, it had a wider translational impact on treatment, institutions and the public profile of the disease. First and foremost, it stimulated the search for other drugs to treat RA and the reassessment of existing treatments, all of which combined to transform the experience of sufferers. Second, its profile and promise helped remake rheumatology, aiding its move from spas and physical methods, to hospitals and medical measures. Thirdly, such developments stimulated rheumatology as an academic specialism, with investment in research and the proliferation of trials, which produced the new corticosteroids in the 1950s and NSAIDs in the 1960s (Dixon 2000). These new drugs moved relatively quickly into clinical practice, in large part because of the translational pathways and protocols that those working on cortisone in the 1950s had created.

## Abbreviations

BMJ: British Medical Journal
BRA: British Rheumatism Association
ERC: Empire Rheumatism Council
IRC: International Congress Rheumatology
JAMA: Journal of the American Medical Association
NA: National Archives (Kew, London)

## Footnotes

1 The *Washington* Post was more cautious. Under the headline ‘New Recipe Eases Pain of Arthritis,’ the report stated that ‘A new hormone compound that shows "significant promise" in the treatment of rheumatoid arthritis was described today in the "proceedings of the staff meeting of the Mayo Clinic."’ (Anon 1949a).

2 We follow an approach similar to that of David Jones uses in his book Broken Hearts (Jones 2013), where he uses published clinical reports and reviews to produce a narrative of the complex development of treatments for cardiac disease. His account is exemplary in demonstrating the pragmatism clinical practice and the many factors shaping decisions.
